# Dietary canthaxanthin improves egg production rate through regulating hepatic lipid metabolism and redox status in indigenous chickens

**DOI:** 10.3389/fvets.2025.1607039

**Published:** 2025-06-05

**Authors:** Jie Chen, Sumeng Yu, Xiaofeng Zhang, Yiping Song, Xiaoyun Zhou, Mei Xiao, Wenchao Liu, Lilong An

**Affiliations:** Department of Animal Science, College of Coastal Agricultural Sciences, Guangdong Ocean University, Zhanjiang, China

**Keywords:** oxidative stress, chickens, canthaxanthin, liver, lipid metabolism, egg production rate

## Abstract

Oxidative stress during egg production disrupts hepatic lipid metabolism and impairs laying performance in hens. This study investigated the effects of dietary canthaxanthin (CX) supplementation (0, 4, 6, 8, 10 mg/kg) on hepatic histomorphology, lipid metabolism, antioxidant capacity, and egg production in indigenous chickens reared under controlled conditions (25 ± 2°C, 65–75% humidity). A total of 180 one-day-old female chickens were randomly assigned to a control group (NC) and four treatment groups (NT1, NT2, NT3, NT4). The trial lasted 9 weeks, with sampling performed at weeks 3, 6, and 9. During the experimental period, compared with the control group, dietary CX supplementation improved the liver weight, egg production rate, serum HDL-C, TG, TC in liver and serum (*p* < 0.05). At week 6, dietary 6 mg/kg CX supplementation significantly reduced yolk TG and serum LDL-C levels (*p* < 0.05), while 10 mg/kg CX significantly increased serum T-AOC and SOD activities (*p* < 0.05). At week 3, 10 mg/kg CX significantly enhanced serum CAT and GSH-Px activities (*p* < 0.05). At week 9, 8 mg/kg CX significantly decreased serum MDA levels (*p* < 0.05). Histological analysis revealed that CX improved liver cell structure, reducing vacuolar degeneration and lipid droplet deposition. Additionally, CX significantly upregulated the expression of SREBP-1c, FASN, ACC, ME, and LXRα in the liver (*p* < 0.05). In conclusion, dietary supplementation with CX demonstrates beneficial effects on lipid metabolism, antioxidant capacity, and egg production in laying hens, with an optimal recommended dosage of 8 mg/kg. This study provides theoretical evidence supporting CX as a functional feed additive to ameliorate lipid metabolic disorders and enhance laying performance in poultry production.

## 1 Introduction

The Huaixiang chicken is an indigenous poultry breed endemic to the western Guangdong region of China. It shows strong stress resistance and efficient lipid metabolism. Its egg quality exceeds commercial breeds. The studies (1) showed that the indigenous chicken could still maintain high liver GSH-Px enzyme activity under harsh environment, and its yolk nutritional value was significantly higher than that of commercial varieties such as Roman brown shell laying hens. However, its egg production is only 40–50% of commercial layer hens such as Hyline Brown. This unique combination of physiological characteristics, namely excellent stress resistance and lipid metabolism, coexists with relatively low egg production rate, making it an ideal animal model for studying the interaction of oxidative stress with lipid metabolism and egg production performance. Stress is the ability of an organism to adapt to external changes. Oxidative stress is prevalent during the egg-laying period. The continuous ovulation of laying chickens will lead to the stress of laying eggs (2) and induce oxidative stress, so the laying cycle is shortened sharply (3). This will have a negative impact on the production performance, egg quality and health of laying chickens. This stress reaction will lead to the accumulation of reactive oxygen species (ROS) in the body, which will induce lipid peroxidation (4), and ultimately affect the stability and quality of lipids in egg yolk. The liver is an important metabolic organ that is responsible for 95% of lipid synthesis and degradation in the body. Normally, the liver converts ingested fat into energy for its own needs or stores it through a series of biochemical reactions. It also regulates lipid transport in the blood to maintain lipid homeostasis in the body. Lipid metabolic activity in the liver directly influences egg yolk formation and lipid content. When oxidative stress occurs in laying chickens, the liver metabolic pathway will be disturbed and lipid metabolism will be disordered, which will directly reduce fat synthesis and affect its ability to store energy (5). Studies have found that ROS can lead to down-regulation of adipogenic genes in 3 T3-L1 adipocytes (6). It also reduces the antioxidant capacity of the chicken liver and increases the content of MDA (7), thereby inducing pathological damage to the liver, mainly manifested as hepatic steatosis (8). These disorders not only affect the health of the liver, but also affect the lipid content of egg yolks through blood transportation and even adversely affect human health. Therefore, the search for effective antioxidants to alleviate the effects of oxidative stress on hepatic lipid metabolism and laying rate in indigenous chickens has become an urgent problem to be solved.

CX is a natural ketocarotenoid. Its chemical name is *β*-carotene-4,4 ‘-dione, and its molecular formula is C_40_H_52_O_2_. It is composed of four isoprene units in a conjugated double bond type connection, and two isoprene units at both ends form a six-membered ring. It contains oxygen functional ketone groups at the C4 and C4 ‘positions of the six-membered ring structure. This special structure gives CX the ability to quench reactive oxygen species and scavenge free radicals (9, 10). The antioxidant capacity of CX is twice that of beta-carotene and fifty times that of vitamin E. Araujo et al. (11) found that the addition of CX to broiler breeders diets increased egg production, performance and reduced feed conversion ratio. Unfortunately, there remains a paucity of research on CX enhancing egg production through hepatic lipid metabolism modulation. This study addresses the issue of oxidative stress-induced lipid metabolic disorders and declining egg production during peak laying periods. We aimed to investigate the effects of dietary CX supplementation on hepatic lipid metabolism, antioxidant capacity, and egg-laying rate in local chickens, thereby providing a scientific basis for enhancing laying hen productivity.

## 2 Materials and methods

### 2.1 Experimental design, birds, canthaxanthin, and diets

The canthaxanthin used in this study was sourced from DSM Vitamin Trading Co., Ltd. (Shanghai, China), with batch number UE01611012 and a 10% canthaxanthin content. A total of 180 one-day-old female Huaixiang chickens (indigenous chickens) were obtained from Guangzhou Xiyin Zhen Breeding Co., Ltd. and raised under standard husbandry conditions (ad libitum access to feed and water, 16:8 h light–dark cycle, ambient temperature maintained at 25 ± 2°C) until 25 weeks of age. They were randomly divided into 5 groups, each with 6 replicate cages (6 chickens per cage, three-layer vertical stepped cage rearing). Each individual cage measuring 40 cm (length) × 52 cm (width) × 38 cm (front height)/33 cm (rear height). Dietary treatments and group assignments are shown in Table 1, and the study lasted 9 weeks. During the testing period, a combination of natural and artificial light was used, maintaining a light intensity of 10–15 Lux for 16 h (16 L:8 D). And the test was conducted at 25 ± 2°C and 65–75% relative humidity, controlled by dehumidifiers and heat lamps. The poultry housing environment was ventilated and disinfected beforehand. A 2-week pre-trial phase (weeks 25–26) standardized conditions, followed by a 9-week formal trial (weeks 27–36, February to April 2022). The experimental diet was a corn-soybean meal basal diet (Table 2), following the “Agricultural Industry Standard of the People’s Republic of China - Chicken Feeding Standard (NY/T 33–2004)” and adapted to local conditions.

**Table 1:** Test groups.

Group	Abbreviation	Daily ration
Control group	NC	Basic diet
Adding Group 1	NT1	Basic diet +4 mg/kg CX
Adding Group 2	NT2	Basic diet +6 mg/kg CX
Adding Group 3	NT3	Basic diet +8 mg/kg CX
Adding Group 4	NT4	Basic diet +10 mg/kg CX

**Table 2:** Basal diet composition and nutrient level (air-dried basis).

Items	Content
Corn	55.00
Soybean meal	20.00
Wheat bran	9.50
Fish meal	5.00
Limestone	7.50
CaHPO_4_	2.50
NaCl	0.10
Premix ^1^	0.40
Total	100.00
ME /(MJ/kg) ^2^	11.60
CP	15.50
Ca	2.0
TP	0.63
Met	0.40
Cys	0.30
Lys	0.80

The nutrient levels are calculated values. The premix provided per kg of diet: VA 9000 IU, VD 2500 IU, VE 20 IU, VB 1212 μg, VK 2.4 mg; Mn100 mg, Zn 60 mg, Fe 25 mg, Cu 5 mg, Co 0.1 mg (Mn, Zn, Fe, Cu, Co are provided in the form of sulphate), Se (N2SeO3•5H2O) 0.2 mg, I (KI) 0.5 mg. All values are measured except metabolizable energy. CaHPO_4_, calcium hydrogen phosphate; NaCl, sodium chloride; ME, metabolic energy; CP, crude protein; Ca, calcium; TP, total phosphorus; Met, methionine; Cys, cystine; Lys, lysine.

### 2.2 Sampling and preservation

During the trial period, egg production was recorded daily at 17:00 to calculate the egg production rate (number of eggs/number of chickens × 100%), with eggs collected daily (7 times per week). On the final two days of the 3rd, 6th, and 9th weeks of the experiment, two eggs per replicate pen were randomly selected, stored at 4°C for ≤24 h, and analyzed for yolk lipid profiles. On the day preceding the 3rd week (30 weeks of age), 6th week (33 weeks of age), and 9th week (36 weeks of age) of the formal trial phase. One chicken was randomly selected from each replicate within every group (6 replicates per group, 2 cages per replicate, 3 chickens per cage, totaling 36 chickens per group). Selected chickens were fasted for 12 h, then weighed to calculate hepatosomatic indices. At 7:00 AM on Sundays of the 3rd, 6th, and 9th experimental weeks, 5 mL of blood was collected from the wing vein of each chicken into centrifuge tubes. Blood was centrifuged at 3000 r/min for 10 min, and serum was separated and stored at-80°C. After blood collection, chickens were euthanized by cervical dislocation. The liver was excised, weighed, and the organ index calculated (organ index = organ mass / body weight × 100%). A 2 × 2 × 2 cm tissue block was taken from the left lower lobe of the liver and fixed with 4% paraformaldehyde to avoid light. Another fresh liver tissue of the same size was fixed in 15% sucrose solution for 6 h, and then transferred to 30% sucrose solution at 4°C overnight. The remaining liver tissues were divided into frozen tubes and stored at-80°C.

### 2.3 Determination of lipid content in serum, liver, and yolk

Parts of serum, liver and egg yolk that had been isolated and stored were used to determine lipid levels. Egg yolk samples were collected from eggs laid during the last two days of experimental weeks 3, 6, and 9, and were isolated on the final collection day using aseptic mechanical separation combined with low-temperature centrifugation. Liver and yolk total cholesterol (TC) and triglyceride (TG) levels were determined by enzymatic colorimetry. Serum TC was measured using anhydrous ethanol extraction, while serum TG was quantified using the GPO-PAP enzymatic method. Serum and yolk low-density lipoprotein cholesterol (LDL-C) and high-density lipoprotein cholesterol (HDL-C) levels were analyzed by ELISA (12). All kits were purchased from Jiangsu Enzyme Immunity Co., Ltd.

### 2.4 Determination of serum antioxidant activity

A part of the serum collected was used to determine the antioxidant parameters. Serum levels of total antioxidant capacity (T-AOC), catalase (CAT), superoxide dismutase (SOD), glutathione peroxidase (GSH-Px), and malondialdehyde (MDA) were determined by ELISA (12). All kits were purchased from Jiangsu Enzyme Immunity Co., Ltd.

### 2.5 Liver HE section and Oil Red O section

In order to observe the damage of liver tissue structure and the accumulation of lipid droplets, HE (hematoxylin–eosin) staining and ORO (Oil Red O) staining were used to make sections. The freshly collected liver tissues were fixed with paraformaldehyde, then sequentially processed through dehydration, clearing, and paraffin infiltration before being embedded in paraffin blocks. Using a Leica microtome, 4 μm-thick sections were obtained and stained with hematoxylin and eosin (HE). The stained sections were examined and photographed under a light microscope equipped with a digital camera system.

Fresh liver tissues were fixed overnight in sucrose solution, embedded in OCT (Optimal Cutting Temperature Compound) compound, and flash-frozen at-20°C. Using a CryoStar NX50 cryostat, 8 μm-thick frozen sections were prepared and stained with Oil Red O (ORO) for lipid visualization. The stained sections were mounted in glycerol gelatin and examined under a light microscope with digital image capture.

### 2.6 Liver lipid-related gene expression

To determine the expression of lipid synthesis-related enzyme genes in the liver, total RNA was extracted using an RNA extraction kit (Nanjing Novozan Biotechnology Co., Ltd.) and reverse-transcribed into cDNA. mRNA levels of sterol regulatory element-binding protein-1c (SREBP-1c), fatty acid synthase (FASN), acetyl-CoA carboxylase (ACC), malic enzyme (ME), and liver X receptor alpha (LXRα) were quantified by qPCR, with *β*-actin as the reference gene. Primers were designed using Primer Premier 5.0 and synthesized by Shanghai Sangon Biotech Co., Ltd. (sequences in Table 3). The qPCR reaction (20 μL) included 1.0 μL cDNA, 0.4 μL each of forward and reverse primers, 10 μL SYBR Green Master Mix, and 8.2 μL nuclease-free water. Cycling conditions: 94°C for 30 s, followed by 40 cycles of 94°C for 5 s, 55°C for 15 s, and 72°C for 10 s. Samples were analyzed in triplicate, and mRNA levels were calculated using the 2^-ΔΔCt^ method.

**Table 3:** Primers for real-time PCR.

Genes	Primer sequence (5′-3′)	Product size (bp)
SREBP-1c	F: GCCCTCTGTGCCTTTGTCTTC R: ACTCAGCCATGATGCTTCTTCC	130
ACACA	F: AATGGCAGCTTTGGAGGTGT R: TCTGTTTGGGTGGGAGGTG	136
FASN	F: CGCAGTTTGTTGATGGTGAG R: TCCTTGGTGTTCGTGACG	179
LXRα	F: GTCCCTGACCCTAATAACCGC R: GTCTCCAACAACATCACCTCTATG	186
ME	F: TGCCAGCATTACGGTTTAGC R: CCATTCCATAACAGCCAAGGTC	175
*β*-actin	F: CAACACAGTGCTGTCTGGTGGTAC R: CTCCTGCTTGCTGATCCACATCTG	199

SREBP-1c, sterol regulatory element-binding protein 1c; ACACA, Ace-CoA carboxylase; FASN, fatty acidsynthase; LXRα, liver X receptor α; ME, malic enzyme.

### 2.7 Statistical analysis

Experimental data were organized using Excel 2021 and analyzed with IBM SPSS 26.0. Results are expressed as means. A two-way ANOVA (period × canthaxanthin) was conducted using the general linear model-univariate method to evaluate main and interaction effects. If no interaction effects were detected, one-way ANOVA was used. Mean differences were assessed by LSD multiple comparisons and Duncan’s test, with significance defined as *p* < 0.05.

## 3 Result

### 3.1 Egg production rate, liver weight and index

The results for laying rate, liver index, and liver weight are presented in Table 4. Canthaxanthin (CX) and experimental period significantly affected laying rate (*p* < 0.05) but had no significant effects on liver index or weight (*p* > 0.05). This indicates that both cantharidin and the time of addition have a significant effect on the laying rate. The interaction of “age ×CX” significantly influenced liver weight and laying rate (*p* < 0.05). This indicates that cantharidin and time of addition together have significant effects on liver weight and laying rate. In Table 4, the addition of 6 mg/kg and 8 mg/kg CX significantly improved laying rate at weeks 6 and 9 (*p* < 0.05), while no significant differences were noted at week 3 (*p* > 0.05). The optimal doses were 8 mg/kg at week 6 and 6 mg/kg at week 9.

**Table 4:** Effect of dietary supplementation with graded levels of canthaxanthin (CX) on liver index, liver weight and egg production rate of laying chickens.

Item	Week	NC	NT1	NT2	NT3	NT4	*P*-value	SEM	*P*-value		
									Period	CX	Period × CX
Liver weight/g	3 weeks	30.51^AB^	28.83^B^	37.17^AB^	41.20^Aa^	26.77^Bb^	0.09	4.04	0.48	0.55	<0.05
	6 weeks	36.03	34.67	28.67	35.00^ab^	35.67^ab^	0.69				
	9 weeks	27.67^B^	26.67^B^	31.33^B^	25.67^Bb^	43.30^Aa^	<0.05				
	*P*-value	0.34	0.36	0.33	<0.05	<0.05					
Liver index/%	3 weeks	1.48	1.22	1.56	1.77	1.23	0.23	0.19	0.07	0.82	0.17
	6 weeks	1.71	1.88	1.62	1.80	1.65	0.86				
	9 weeks	1.56	1.43	1.81	1.46	2.03	0.16				
	*P*-value	0.69	0.06	0.62	0.38	0.02					
Egg production rate%	3 weeks	43.28^ABc^	43.64^ABb^	46.20^Ac^	47.89^Ac^	39.17^Bb^	<0.05	1.77	<0.05	<0.05	<0.05
	6 weeks	49.48^Bb^	48.06^Bb^	55.34^Ab^	55.70^Ab^	39.75^Cb^	<0.05				
	9 weeks	56.09^Ba^	68.77^Aa^	69.89^Aa^	67.07^Aa^	55.34^Ba^	<0.05				
	*P*-value	<0.05	<0.05	<0.05	<0.05	<0.05					

Uppercase letters indicate differences between different dose groups within the same period, while lowercase letters indicate differences within the same dose group across different periods. Data labels without letters or with the same letters indicate no significant difference (*p* > 0.05), whereas different lowercase or uppercase letters denote significant differences (*p* < 0.05).

**Figure 1:**
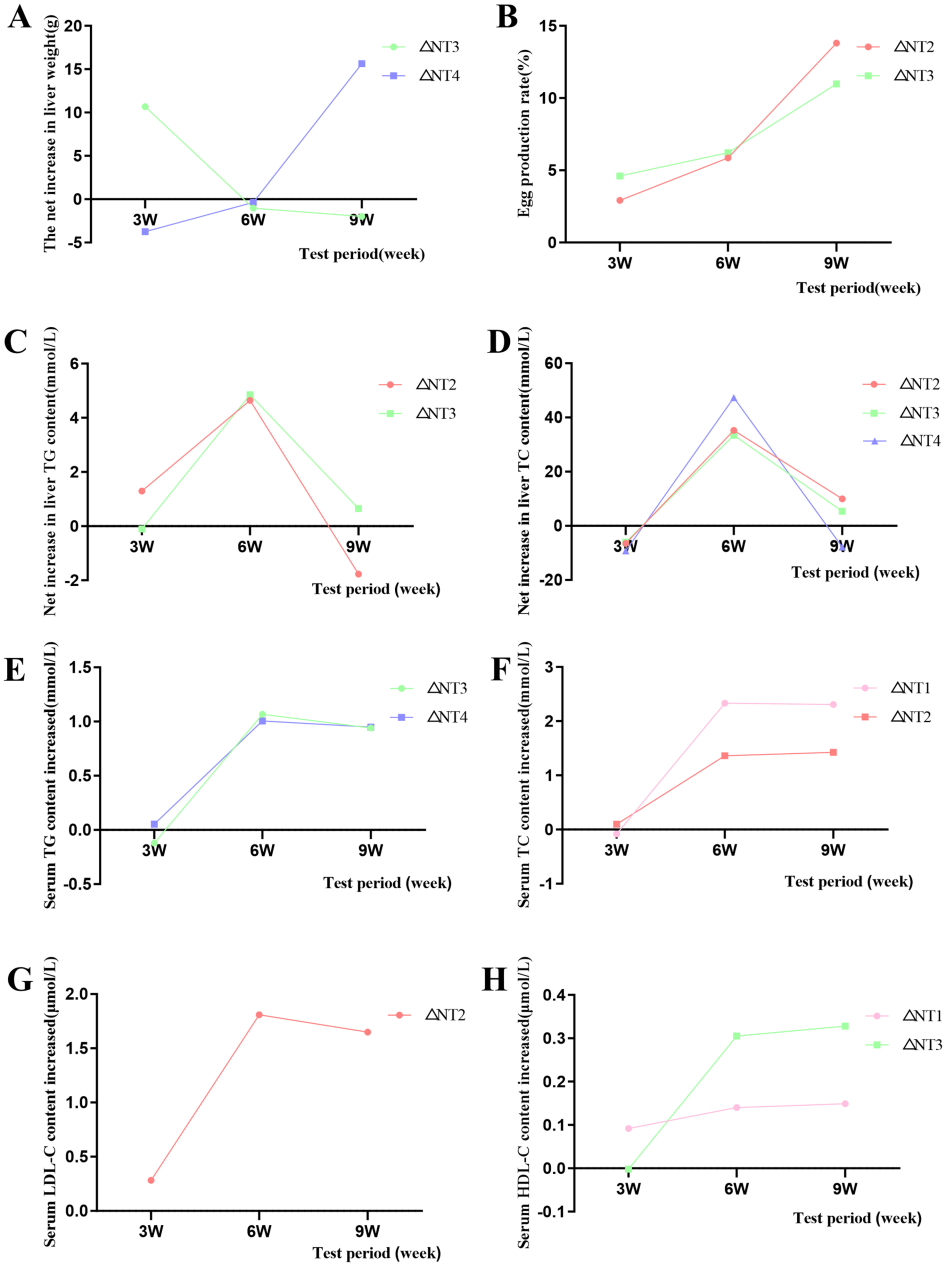
Effects of dietary canthaxanthin on laying rate, liver weight, serum lipids, and hepatic lipid content in Huaixiang Chickens, (n 6/group). A all figures above depict the net increase in each parameter, calculated as the difference between the optimal dose of canthaxanthin and the NC group at each time pomt. **(A)** Liver weight. **(B)** Egg production rate. **(C)** Liver TG, triglyceride. **(D)** Liver TC, total cholesterol. **(E)** Serum TG. **(F)** Serum TC. **(G)** Serum LDL-C lipoprotein cholesterol. **(H)** Serum HDL-C, high-density lipoprotein cholesterol.

Under the optimal dose, the egg production rate showed an increasing trend, and the net increment was positive (Figure 1B), indicating that CX had a significant promoting effect on the improvement of laying rate. For liver weight, at week 9, 10 mg/kg CX significantly increased liver weight compared to the NC group (*p* < 0.05), with no significant differences at weeks 3 and 6 (*p* > 0.05). For liver weight, the optimal doses at week 3, 6, and 9 were 8 mg/kg, 10 mg/kg, and 10 mg/kg, respectively (Figure 1A). Under the optimal dose, liver weight decreased first and then increased with time, and the net increment was positive, which indicated that CX had a significant promoting effect on liver weight. At the 3rd, 6th and 9th week of the experiment, compared with NC group, dietary addition of CX had no significant difference in liver index (*p* > 0.05).

### 3.2 Liver organization and lipid droplet deposition

**Figure 2:**
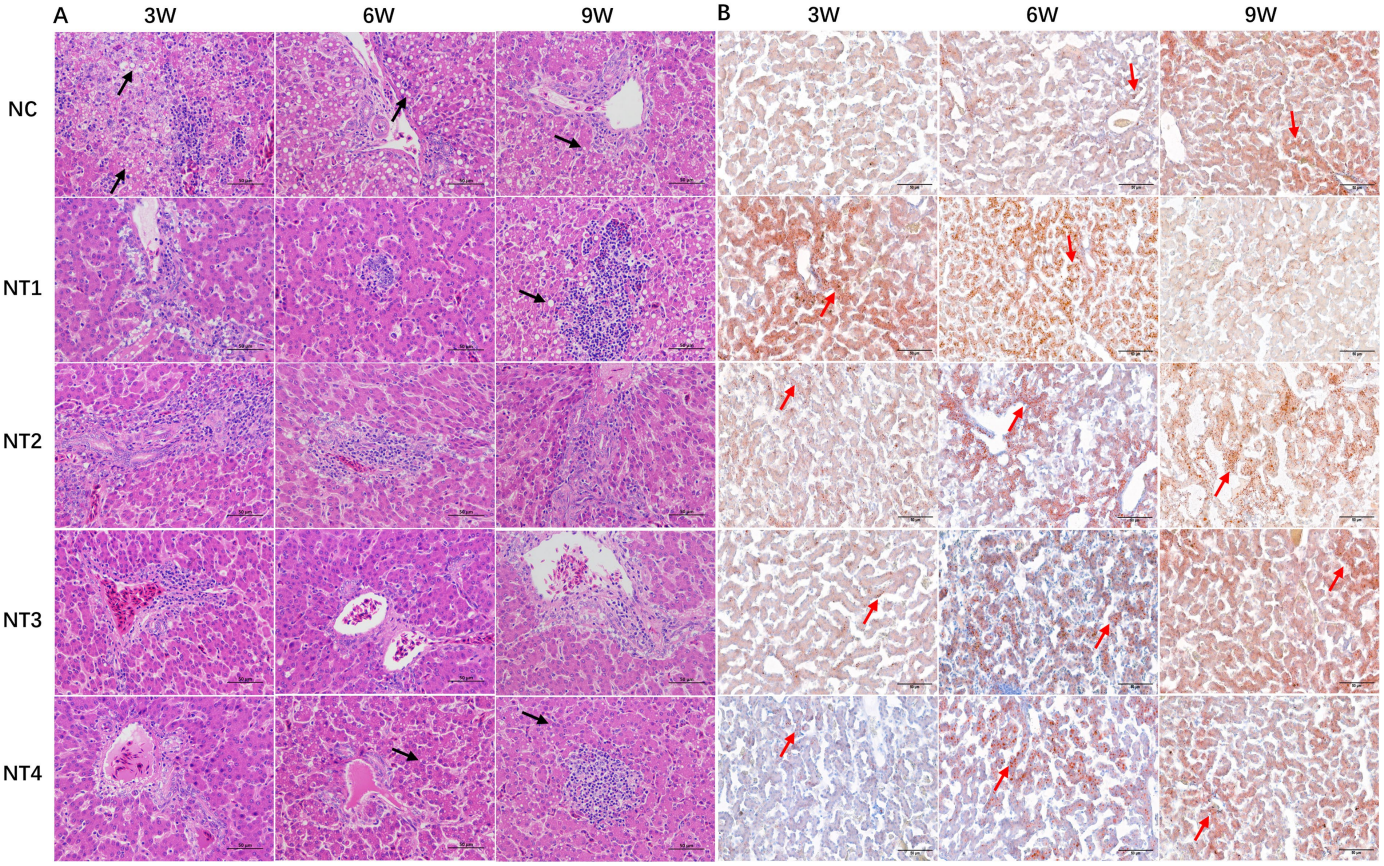
Effects of dietary canthaxanthin on liver histology and lipid droplet depositionin Huaixiang Chickens. (n6/group). The black arrows in the figure indicate cavitation degeneration, and the red arrows represent lipid droplets. **(A)** HE staining of chicken liver tissue (HE×400). **(B)** Oil Red O staining of chicken liver tissue (Oil Red Ox400).

The H&E staining results of the liver are shown in Figure 2A. At week 3, NC group hepatocytes were swollen and rounded, with cytoplasmic vacuoles of varying sizes (black arrows), showing severe vacuolar degeneration, narrowed hepatic sinusoids, nuclear displacement, and disorganized hepatic cords, along with inflammatory infiltration. Compared to NC, 4, 6, and 10 mg/kg CX groups had reduced vacuolar degeneration but still showed inflammation, while 8 mg/kg CX group exhibited the best recovery, with clear hepatocyte structure and organized hepatic cords. At week 6, 10 mg/kg CX group showed vacuolar degeneration, 4 and 6 mg/kg CX groups had inflammation, and 8 mg/kg CX group maintained optimal improvement. By week 9, 4 and 10 mg/kg CX groups had aggravated vacuolar degeneration, 6 mg/kg CX group still showed inflammation, and 8 mg/kg CX group had no pathological changes. The results of Oil Red O Staining are shown in Figure 2B. At week 3, NC group had fewer red-stained particles and lipid droplets, while all CX groups showed significant increases (red arrows), with 4 mg/kg CX group showing the best lipid reduction. At week 6, 4 and 6 mg/kg CX groups had more red-stained particles than NC. By week 9, all CX groups showed reduced red-stained particles and lipid droplets, with 4 mg/kg CX group showing the most significant improvement.

### 3.3 Liver lipid content

The results of liver TG and TC content are shown in Table 5. CX and experimental period had significant main effects on liver TG and TC content (*p* < 0.05), and the interaction of “period × CX” was also significant (*p* < 0.05). This shows that both CX and the duration of supplementation collectively and significantly influence the hepatic TG and TC levels. For liver TG, at week 3, 4 mg/kg CX significantly increased liver TG compared to the NC group (*p* < 0.05). At week 6, all CX groups showed significantly higher liver TG (*p* < 0.05), with 6 mg/kg CX being the most effective. At week 9, no significant differences were observed between CX groups and the NC group (*p* > 0.05). The optimal doses were 4 mg/kg at week 3 and 6 mg/kg at week 6 (Figure 1B). For liver TC, at week 6, all CX groups had significantly higher liver TC than the NC group (*p* < 0.05), with 10 mg/kg CX being the most effective. At weeks 3 and 9, no significant differences were observed between CX groups and the NC group (*p* > 0.05). For hepatic TC, the optimal doses at week 6 and week 9 is 10 mg/kg and 6 mg/kg, respectively (Figure 1D). Under the optimal dose, the contents of TG and TC showed an initial increase followed by a decrease over time. The net increases were positive, indicating CX had a promoting effect.

**Table 5:** Effect of dietary supplementation with graded levels of canthaxanthin (CX) on serum and liver lipid content of laying chickens.

Item	Week	NC	NT1	NT2	NT3	NT4	*P*-value	SEM	*P*-value		
									Period	CX	Period × CX
Liver TG/mmol/L	3 weeks	6.45^Bb^	7.74^Ac^	6.34^Bb^	6.3219^Bb^	5.97^Bb^	<0.05	0.32	<0.05	<0.05	<0.05
	6 weeks	6.32^Bb^	10.96^Ab^	11.17^Ab^	10.49^Aa^	10.85^Aa^	<0.05				
	9 weeks	10.79^ABa^	9.01^Ca^	11.43^Ab^	10.05^Ba^	10.69^ABa^	<0.05				
	*P*-value	<0.05	<0.05	<0.05	<0.05	<0.05					
Liver TC/mmol/L	3 weeks	81.30^Ac^	59.59^Bc^	53.11^Bc^	53.58^Bc^	50.35^Bc^	<0.05	4.22	<0.05	0.07	<0.05
	6 weeks	125.75^Cb^	124.06^Cb^	159.30^Bb^	157.53^Ba^	171.34^Aa^	<0.05				
	9 weeks	153.85^ABa^	150.663^Aa^	160.61^Aa^	156.05^Ab^	142.82^Bb^	0.05				
	*P*-value	<0.05	<0.05	<0.05	<0.05	<0.05					
Serum TG/mmol/L	3 weeks	2.33^a^	2.36^b^	2.22^b^	2.21^b^	2.39^b^	0.47	0.08	<0.05	<0.05	<0.05
	6 weeks	1.98^Cb^	2.67^Ba^	2.63^Ba^	3.05^Aa^	2.99^Aa^	<0.05				
	9 weeks	1.88^Cb^	2.69^ABa^	2.58^Ba^	2.82^Aa^	2.83^Aa^	<0.05				
	*P*-value	<0.05	<0.05	<0.05	<0.05	<0.05					
Serum TC/mmol/mL	3 weeks	5.55	5.47^b^	5.65^b^	5.08^b^	5.31^b^	0.57	0.26	<0.05	<0.05	<0.05
	6 weeks	6.28^C^	8.61^Aa^	7.64^Ba^	8.20^ABa^	7.60^Ba^	<0.05				
	9 weeks	6.22^C^	8.53^Aa^	7.64^Ba^	8.23^ABa^	7.48^Ba^	<0.05				
	*P*-value	0.11	<0.05	<0.05	<0.05	<0.05					
Serum LDL-C/μmol/L	3 weeks	2.75^Ab^	2.19^Ab^	2.17^Ab^	2.08^Bb^	2.92^Ab^	<0.05	0.25	<0.05	<0.05	<0.05
	6 weeks	3.55^Aa^	3.47^Aa^	2.36^Bb^	3.15^Aa^	3.401^Ab^	<0.05				
	9 weeks	3.22^Bab^	4.00^Aa^	3.53^ABa^	2.41^Cb^	4.09^Aa^	<0.05				
	*P*-value	<0.05	<0.05	<0.05	<0.05	<0.05					
Serum HDL-C/μmol/L	3 weeks	1.77^Aa^	1.86^Aa^	1.65^Ba^	1.77^Aa^	1.61^Ba^	<0.05	<0.05	<0.05	<0.05	<0.05
	6 weeks	0.83^Cb^	0.97^Bb^	1.04^ABb^	1.14^Ab^	1.04^ABb^	<0.05				
	9 weeks	0.78^Cb^	0.93^Bb^	0.99^Bb^	1.11^Ab^	0.98^Bb^	<0.05				
	*P*-value	<0.05	<0.05	<0.05	<0.05	<0.05					

Uppercase letters indicate differences between different dose groups within the same period, while lowercase letters indicate differences within the same dose group across different periods. Data labels without letters or with the same letters indicate no significant difference (*P* > 0.05), whereas different lowercase or uppercase letters denote significant differences (*P* < 0.05).

### 3.4 Serum lipid content

Serum lipid content results are shown in Table 5. CX and experimental period significantly affected serum TG, TC, LDL-C, and HDL-C (*p* < 0.05). The “period × CX” interaction was also significant (*p* < 0.05). This shows that both CX and the duration of supplementation collectively and significantly influence the serum TG, TC, LDL-C and HDL-C contents. At weeks 6 and 9, 8 mg/kg CX significantly increased serum TG and HDL-C (*p* < 0.05) and reduced LDL-C (*p* < 0.05). Meanwhile, 4 mg/kg CX significantly increased serum TC (*p* < 0.05). At week 6, 6 mg/kg CX significantly reduced serum LDL-C (*p* < 0.05). For serum TG, the optimal doses were 10 mg/kg at week 3, 8 mg/kg at week 6, and 10 mg/kg at week 9 (Figure 1E). For serum TC, the optimal doses were 6 mg/kg at week 3, 4 mg/kg at week 6, and 4 mg/kg at week 9 (Figure 1F). For serum LDL-C, the optimal doses were 8 mg/kg at week 3, 6 mg/kg at week 6, and 8 mg/kg at week 9 (Figure 1G). For serum HDL-C, the optimal doses were 4 mg/kg at week 3, 8 mg/kg at week 6, and 8 mg/kg at week 9 (Figure 1H). Under the optimal dose, the contents of TC, LDL-C and HDL-C in serum increased with the test time, while the contents of TG in serum increased first and then decreased. Under the optimal dose, compared to the NC group, the net increment of TG, TC and HDL-C in serum was positive, and the net increment of LDL-C in serum was negative, indicating a cumulative effect of CX.

### 3.5 Egg yolk lipid content

**Figure 3:**
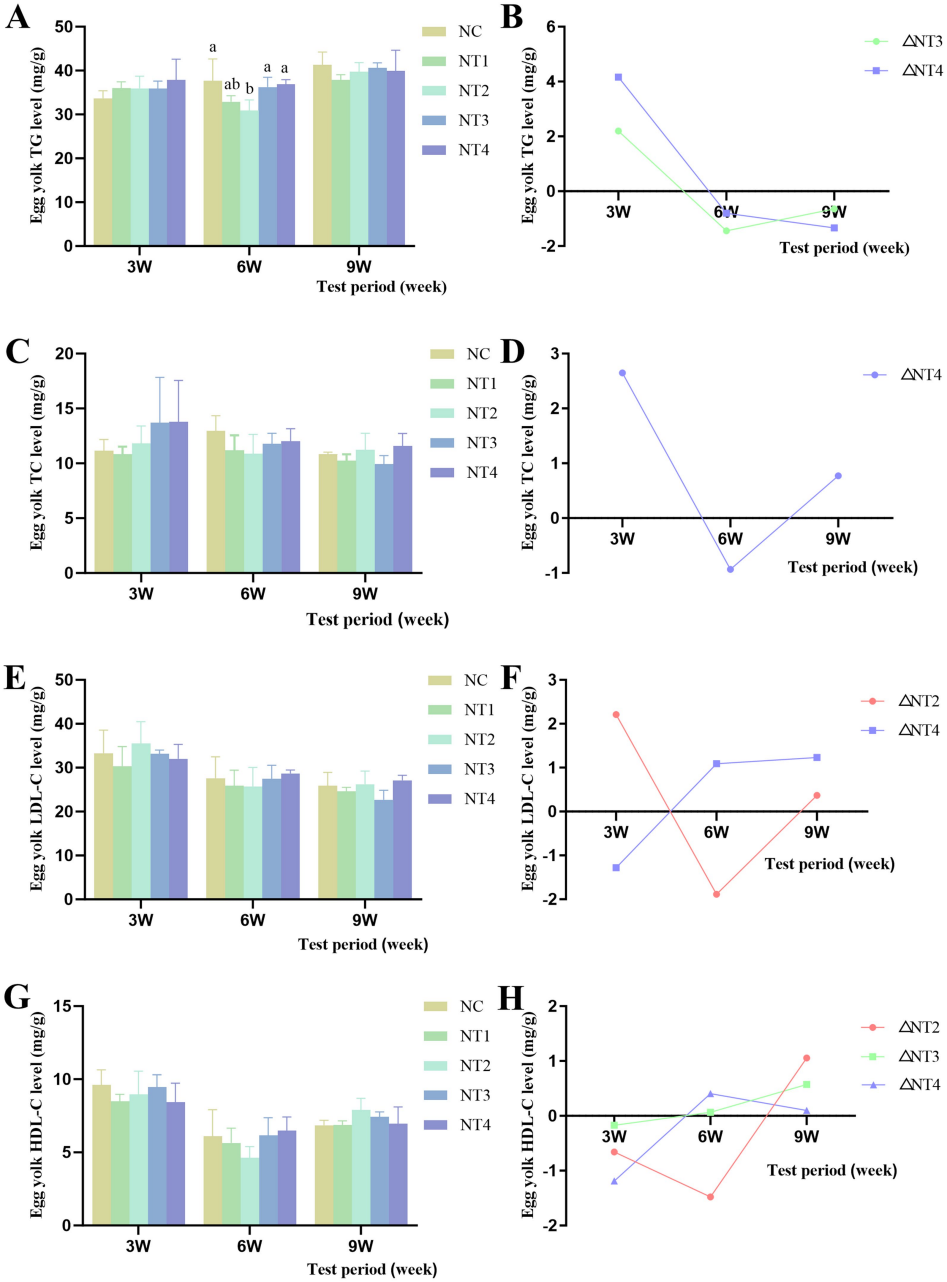
Effects of dietary canthaxanthin on yolk lipid contentin Huaixiang Chickens. (n6/group). A all figures above depiet the net increase in each parameter, calculated as the difference between the optimal dose of canthaxanthin and the NC group at each time point. **(A)** Egg yolk TG levels, triglyceride. **(B)** Net increase in TG within the yolk. **(C)** Egg yolk TC levels, total cholesterol. **(D)** Net increase in TC within the yolk. **(E)** Egg yolk 1. D1.-C levels, lipoprotein cholesterol. **(F)** Net increase in L. DI.-C within the yolk. **(G)** Figg yolk HDI.-C levels,high-density lipoprotein cholesterol. **(H)** Net increase in HDL-C within the yolk.

The results of yolk lipid content are shown in Figure 3. Compared to the NC group, CX had no significant effect on yolk TG, TC, LDL-C, or HDL-C levels (*p* > 0.05). However, at week 6, 6 mg/kg CX significantly reduced yolk TG content (*p* < 0.05). Throughout the trial, under the optimal dose, the net increase in yolk TG levels showed a declining trend after adding the optimal dose of CX (Figure 3B), indicating that CX had no significant improvement or cumulative effect on yolk TG. In contrast, the net increase in yolk TC levels exhibited an initial decrease followed by an increase (Figure 3D), while yolk LDL-C levels showed a gradual decline with positive net increases (Figure 3F). Yolk HDL-C levels demonstrated an upward trend with positive net increases (Figure 3H), suggesting that CX had cumulative effects on yolk TC, LDL-C, and HDL-C.

### 3.6 Serum antioxidant capacity

The serum antioxidant capacity results are presented in Table 6. Both experimental period and CX significantly affected serum T-AOC, MDA, CAT, and GSH-Px activities (*p* < 0.05). And CX had significant main effects on serum T-AOC, MDA, CAT, GSH-Px and SOD (*p* < 0.05). Moreover, the interaction of “period ×CX” on serum T-AOC and MDA was significant (*p* < 0.05). These results indicated that CX and the addition time could affect the content of T-AOC and MDA in serum. At week 3, dietary supplementation with 4, 6, 8, and 10 mg/kg CX significantly increased serum CAT, GSH-Px, and SOD activities (*p* < 0.05). At weeks 6 and 9, supplementation with 4, 6, 8, and 10 mg/kg CX significantly increased serum T-AOC, CAT, GSH-Px, and SOD activities (*p* < 0.05) and reduced serum MDA levels (*p* < 0.05). The 8 and 10 mg/kg CX doses showed the best effects. The optimal doses for serum T-AOC were 6 mg/kg at week 3, 10 mg/kg at week 6, and 10 mg/kg at week 9. For serum MDA, the optimal doses were 4 mg/kg at week 3, 8 mg/kg at week 6, and 8 mg/kg at week 9. For serum CAT, the optimal doses were 10 mg/kg at week 3, 8 mg/kg at week 6, and 8 mg/kg at week 9. For serum GSH-Px, the optimal doses were 10 mg/kg at week 3, 4 mg/kg at week 6, and 10 mg/kg at week 9. Additionally, 10 mg/kg CX consistently enhanced serum SOD activity throughout the trial. During the mid-to-late trial period, under the optimal dose of CX, compared with that in the NC group, the net increment of serum antioxidant capacity (T-AOC, CAT, GSH-Px and SOD enzyme activity) was positive, and the serum MDA content was negative, which indicated that CX had a promoting effect on improving the antioxidant capacity of the body.

**Table 6:** Effect of dietary supplementation with graded levels of canthaxanthin (CX) on serum antioxidant properties of laying chickens.

Item	Week	NC	NT1	NT2	NT3	NT4	*P*-value	SEM	*P*-value		
									Period	CX	Period × CX
T-AOC (μmolTrolox/g)	3 weeks	3.55^ABa^	3.23^Bb^	3.59^Ab^	3.59^Ab^	3.38^Ab^	0.15	0.12	<0.05	<0.05	<0.05
	6 weeks	2.78^Bb^	4.134^Aa^	4.08^Aa^	4.14^Aa^	4.32^Aa^	<0.05				
	9 weeks	2.84^Bb^	4.06^Aa^	4.00^Aa^	4.27^Aa^	4.31^Aa^	<0.05				
	*P*-value	<0.05	<0.05	<0.05	<0.05	<0.05					
MDA (nmol/L)	3 weeks	11.70^Bb^	13.75^Ab^	13.95^Ab^	14.32^Ab^	14.37^Ab^	<0.05	0.36	<0.05	<0.05	<0.05
	6 weeks	20.40^Aa^	19.03^Ba^	18.20^BCa^	17.43^Ca^	18.26^BCa^	<0.05				
	9 weeks	20.39^Aa^	19.14^Ba^	17.83^CDa^	16.97^Da^	18.31^BCa^	<0.05				
	*P*-value	<0.05	<0.05	<0.05	<0.05	<0.05					
CAT (U/mL)	3 weeks	43.65^Da^	49.44^Ca^	52.07B^Ca^	52.61^ABa^	55.67^Aa^	<0.05	1.08	<0.05	<0.05	0.12
	6 weeks	38.00^Cb^	43.35^Bb^	46.46^ABb^	49.14^Ab^	45.10^Bb^	<0.05				
	9 weeks	37.36^Db^	43.49^Cb^	46.77^ABb^	49.21^Ab^	45.58^BCb^	<0.05				
	*P*-value	<0.05	<0.05	<0.05	<0.05	<0.05					
GSH-Px (IU/L)	3 weeks	459.53^C^	536.39^B^	559.70^ABa^	559.41^AB^	589.31^A^	<0.05	15.26	<0.05	<0.05	0.63
	6 weeks	412.18^B^	537.77^A^	507.59^Ab^	513.67^A^	522.26^A^	<0.05				
	9 weeks	411.04^B^	522.76^A^	501.63^Ab^	509.32^A^	532.01^A^	<0.05				
	*P*-value	0.051	0.75	<0.05	0.05	0.01					
SOD (U/mL)	3 weeks	88.21^D^	102.43^C^	105.15^BC^	110.42^AB^	113.04^A^	<0.05	1.87	0.46	<0.05	0.26
	6 weeks	84.41^B^	105.49^B^	107.66^AB^	104.27^B^	111.55^A^	<0.05				
	9 weeks	84.15^C^	103.84^B^	107.80^AB^	105.28^B^	111.19^A^	<0.05				
	*P*-value	0.25	0.52	0.54	0.06	0.76					

Uppercase letters indicate differences between different dose groups within the same period, while lowercase letters indicate differences within the same dose group across different periods. Data labels without letters or with the same letters indicate no significant difference (P > 0.05), whereas different lowercase or uppercase letters denote significant differences (*P* < 0.05).

### 3.7 Expression of hepatic lipid synthesis-related enzyme genes

**Figure 4:**
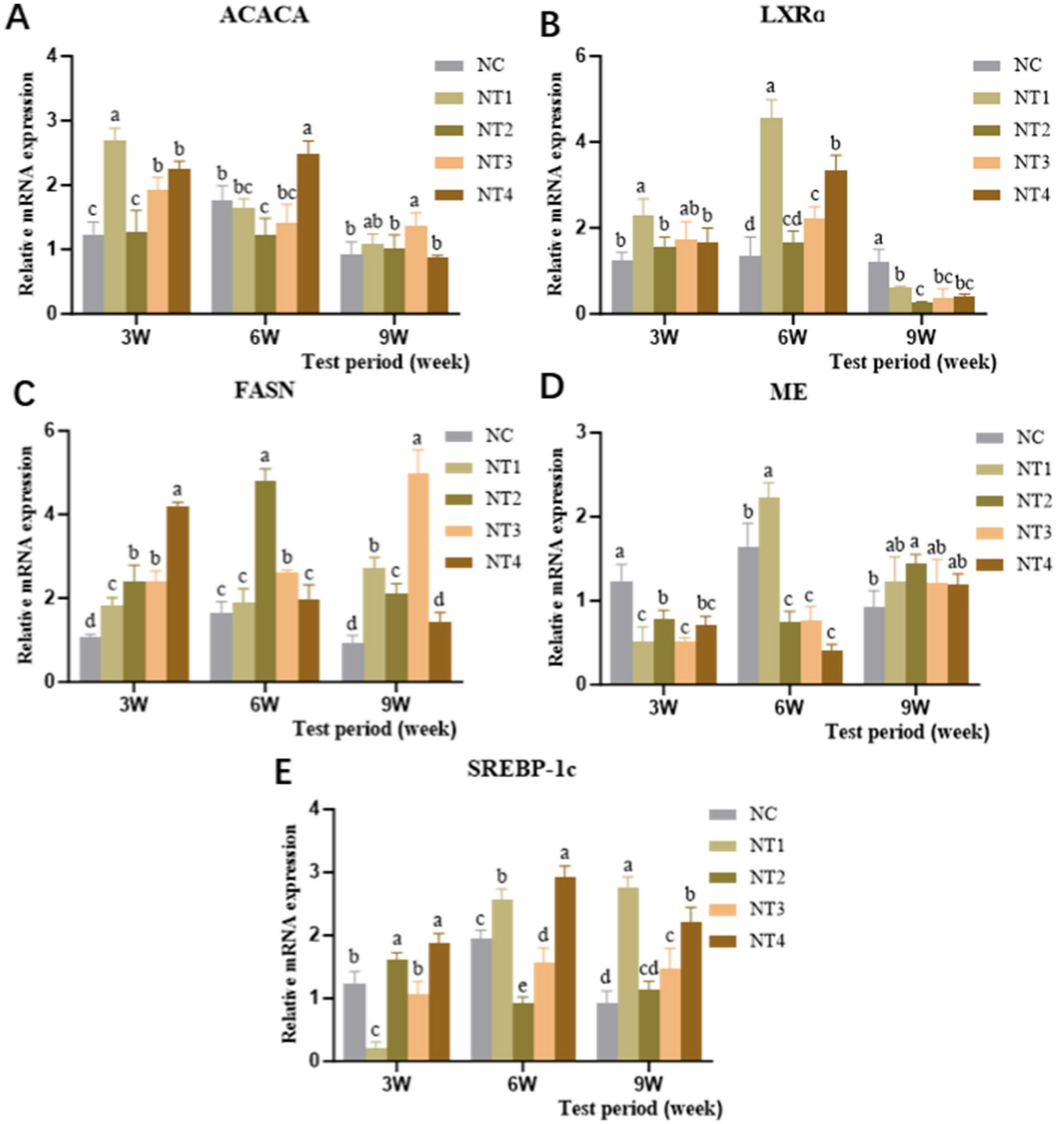
Effects of dietary canthaxanthin on the relative mRNA expression of hepatic lipid synthesis-related enzyme genes in Huaixiang Chickens. (*n* = 6/group). **(A)** ACACA, Ace-CoA carboxylase. **(B)** LXRa, liver X receptor. **(C)** FASN, fatty acid synthetase. **(D)** ME, malidase. **(E)** SREBP-1c, sterol regulatory element binding protein 1c.

The relative mRNA expression levels of lipid synthesis-related enzyme genes in the liver are shown in Figure 4. At week 3, compared to the NC group, dietary supplementation with 4 mg/kg CX significantly increased the mRNA expression of ACACA and LXRα genes (*p* < 0.05). Meanwhile, 6 and 10 mg/kg CX significantly upregulated the mRNA expression of FASN and SREBP-1c genes (*p* < 0.05). At week 6, 10 mg/kg CX significantly enhanced the mRNA expression of ACACA and SREBP-1c genes (*p* < 0.05), while 4 mg/kg CX significantly increased the mRNA expression of ME and LXRα genes (*p* < 0.05). Additionally, 6 and 8 mg/kg CX significantly elevated the mRNA expression of the FASN gene (*p* < 0.05). At week 9, 8 mg/kg CX significantly boosted the mRNA expression of the ACACA gene (*p* < 0.05). The FASN gene expression in the 4, 6, and 8 mg/kg CX groups was significantly higher than in the NC group (*p* < 0.05). Furthermore, 6 mg/kg CX significantly upregulated the mRNA expression of the ME gene (*p* < 0.05), and the 4, 8, and 10 mg/kg CX groups showed significant upregulation of SREBP-1c gene expression (*p* < 0.05). Overall, dietary supplementation with CX led to an upward trend in the expression of ACACA, FASN, ME, and SREBP-1c genes. The results showed that CX could promote the expression of lipid synthesis-related enzyme genes and had a cumulative effect.

## 4 Discussion

Huaixiang chicken is an excellent indigenous chicken breed in China. It has delicious meat and strong stress resistance, but its egg production performance is relatively low (13). The annual egg production is only 120–150 eggs (14), which is significantly lower than the 280–320 eggs of commercial laying hen breeds, mainly due to the insufficient development efficiency of follicles caused by its genetic background. During the peak egg production period, Huaixiang chickens are prone to oxidative stress (3), which will interfere with the lipid metabolism of the liver. This metabolic disturbance is characterized by reduced GSH-Px activity and downregulated expression of lipid synthesis-related genes such as PPAR-*γ* and FASN (15), ultimately affecting the egg production sustainability (16). In addition, as the main site for fat synthesis and degradation in laying hens, the liver plays a unique role in lipid metabolism. Approximately 90% of fat is synthesized in the liver, with the remaining 10% derived from adipose tissue (17–20). The results of this experiment showed that the liver oxidative stress injury of chickens during laying period was serious. H&E staining revealed extensive vacuolar degeneration of hepatocytes, disrupted liver cell structures accompanied by inflammatory infiltration, and reduced liver weight, consistent with the findings of Baéza et al. (21). ROS can induce fat infiltration and fatty liver in poultry, leading to liver dysfunction, increased liver index, and weight loss. Li et al. (22) found that ROS-mediated chronic DEHP exposure caused central venous congestion, hepatic sinusoid dilation, and hepatocyte nuclear dissolution in broiler liver tissues. While normal redox reactions promote the growth and egg production of laying hens, during peak laying periods, the high energy demand for yolk formation increases oxygen consumption. Liver lipid synthesis becomes crucial for yolk formation but also places additional burden on the liver. Excessive ROS accumulation in the liver (23) disrupts the oxidative-antioxidant balance, attacks hepatocytes, and induces oxidative stress damage, affecting liver structure, lipid synthesis, and transport functions. This is likely the primary mechanism by which oxidative stress causes liver damage.

In recent years, CX has been recognized as a natural antioxidant used to alleviate oxidative stress in laying hens, which scavenges free radicals in the organism (17), improves laying performance (18) and antioxidant capacity, and mitigates the negative effects of stress (19). In this experiment, the hepatocytes in the CX group had a clear structure, no swelling and rounding, reduced vacuolar degeneration, and tightly arranged hepatic cords, with 8 mg/kg CX being the most effective. As the duration of the trial increased, CX was most effective in repairing liver structure by week 9 of the trial, suggesting that CX has a cumulative effect and that it continues to work in the organism. Jiarui et al. (20) found that the addition of CX to the diet was effective in alleviating liver tissue injury and inflammatory response in broilers. It has been reported (24) that carotenoids can prevent hepatic steatosis in an obese mouse model. It acts directly on hepatocytes (25) by reducing lipid accumulation, enhancing insulin signaling and inhibiting inflammatory signaling pathways to regulate liver health. Therefore, the results of this experiment showed that CX can be used as a feed additive to ameliorate pathological liver injury. In addition, during the growth of laying hens, with the gradual increase in feed intake and body weight, in order to meet the demand for egg production, the liver must grow rapidly to form a certain ratio with body weight in order to maintain normal material transportation and metabolism (26). Liver index and weight are important indicators of the liver health of laying hens (27). The results of this experiment showed that oxidative stress resulted in a general decrease in liver weight of laying hens in the NC group. However, CX was able to significantly increase liver weight but had no significant effect on liver index, which may be related to the lower body weight of laying hens. The addition of 8 mg/kg CX at week 3 and 10 mg/kg CX at weeks 6 and 9 of the experiment promoted liver development and showed a cumulative effect. This indicates that the addition of different doses of CX to the diet throughout the experimental period was able to gradually promote liver growth, which is consistent with the normal growth and developmental pattern of the organism.

The liver, as the central organ of lipid metabolism (28), is responsible for synthesizing TG, TC, and phospholipids. These lipids bind to apolipoproteins to form low-density lipoprotein (LDL), intermediate-density lipoprotein (IDL), high-density lipoprotein (HDL), and vitellogenin (VTG), which are transported under estrogen regulation to oocytes via the bloodstream to participate in yolk formation (29, 30), playing a critical role in the reproductive performance of laying hens (31). Poultry ovaries lack the ability to autonomously synthesize lipids; thus, yolk lipid deposition entirely relies on hepatic metabolism and plasma transport, indirectly reflecting the liver’s lipid metabolic status. Laying hens need glucose for liver lipid synthesis. Glucose comes from dietary carbohydrate metabolism (32). It undergoes complex reactions to synthesize fatty acids. Studies show glucose-derived pyruvate enters the citric acid cycle (33). It generates apolipoproteins and phospholipids. These wrap around TG and TC to form VLDL. Acetyl-CoA converts to malonyl-CoA. This participates in fatty acid synthesis. FA interacts with ACS isoenzymes. It forms acyl-CoA for *de novo* fat synthesis and *β*-oxidation. Key enzymes like malate dehydrogenase are activated. Fatty acid synthase is also activated. They promote TG and TC synthesis (34, 35). These lipids are then transported into the blood through complex assembly. So the levels of TG and TC in the blood reflect the lipid metabolism of the organism. In addition, the dynamic balance of LDL-C and HDL-C reflects the reverse cholesterol transport capacity and the metabolic state of the liver. Oxidative stress inhibits lipid synthase activity. It increases lipid breakdown. This reduces TG and TC synthesis. Blood levels of TG, TC, HDL-C, and LDL-C decrease. Their transport and metabolism also decline. This hinders yolk formation. It ultimately affects egg production rate. Liu et al. (36) found that under oxidative stress conditions, fish plasma TC, TG and free fatty acid levels were reduced, and the activities of various metabolic enzymes such as ACC and FAS were significantly reduced. Zhang et al. (37) reported that ROS overproduction affects the balance of lipid synthesis and catabolism, which in turn leads to the blockage of hepatic lipid synthesis, which is consistent with our experimental results. CX is a fat-soluble antioxidant and a free radical scavenger (38). Linseisen et al. (39) found that CX deposition protects LDL-C from oxidation. Palozza et al. (40)found that *in vitro* that CX inhibits ROS-induced TC oxidation and has anti-atherosclerotic effects. It is hypothesized that CX may promote the synthesis of TG and total TC in the liver by enhancing the activity of lipid synthesis enzymes, and then improve the lipid transport efficiency to support the formation of egg yolks (13). The results of this study showed that compared with NC group, CX could significantly increase the levels of TG and TC in liver and the levels of TG, TC and HDL-C in serum of chickens during laying period, and reduce the level of HDL-C in serum. This accelerates the rate of lipid synthesis in the liver and metabolic transport in the blood. In the 6th week of the experiment, adding 8 and 10 mg / kg CX to the diet was beneficial to promote the synthesis of TG and TC in the liver. In terms of increasing serum TG, TC, HDL-C content and reducing LDL-C, the net value added of the optimal dose of CX in each period was positive and showed an increasing trend. The results showed that CX played a positive role in regulating serum TG, TC, HDL-C and LDL-C levels throughout the experimental period, with a cumulative effect. We speculate that CX may enhance lipid synthase activity. This promotes liver TG and TC synthesis. It accelerates the transport of TG, TC, HDL-C, and LDL-C in blood. More lipids are provided to ovarian tissues. This supports yolk formation. These findings align with previous research (41). However, this study found that CX did not significantly promote lipid deposition in egg yolks. Levels of TG, TC, HDL-C, and LDL-C in the yolks showed no notable increase. Yet, the egg production rate rose. It is speculated that CX might enhance liver lipid synthesis. It could also accelerate lipid transport to oocytes. This may increase the number of yolks formed. It does not raise the lipid content per yolk. Thus, egg production rate improves.

To further investigate the effects of CX on liver lipid synthesis, we observed lipid droplet distribution using Oil Red O staining. The results showed that the NC group had fewer lipid droplets at weeks 3 and 6, but an increase was observed at week 9. Studies have shown that hepatic lipid droplets (LD) are not only involved in fat mobilization and storage, but also play an important role in cellular antioxidation (42). Under oxidative stress, LD activate a lipid protective mechanism, autonomously positioning themselves to shield the unsaturated fatty acids in the TG core from oxidation, thereby maintaining hepatic lipid metabolic homeostasis (43–45). After oxidative stress subsides, lipid droplets gradually dissipate. This process involves many lipid droplet-related proteins (46). Lipopolysaccharide-binding protein (LBP) and antioxidant protein (PRDX4) are molecular chaperones (37). They work together to regulate LD. This prevents lipid peroxidation. PRDX4 acts as an oxidative sensor for LBP, ensuring the free movement of LBP into and out of LD, while LBP transports PRDX4 into LD to protect it and ensure its proper function (37). The results of this experiment showed that at weeks 3 and 6, the CX-supplemented groups had more red-stained particles. Large red-stained areas were observed in the 4 and 6 mg/kg groups. It is speculated that excessive ROS accumulation may have damaged LBP and PRDX4. CX likely stimulated lipid droplet-associated proteins to activate the lipid protection mechanism. This led to lipid droplet deposition, preventing oxidative damage. By week 9, lipid droplet content decreased in all CX supplementation groups. The 4 mg/kg CX group showed the best results. This indicates that low-dose CX can gradually alleviate liver oxidative stress. It also produces a cumulative effect in the body. From initially activating the lipid protective mechanism to eventually resolving it, CX played a positive regulatory role throughout the process.

The continuous increase in serum MDA concentration in the NC group throughout the trial period indicated that laying hens experienced oxidative stress. This led to intensified lipid peroxidation in the body. The result is consistent with the oxidative damage shown in liver HE staining. During peak laying periods, high levels of ROS accumulate in laying hens due to continuous egg production. Physiological oxidative levels support normal oocyte growth and development. However, prolonged high oxidative states can trigger severe oxidative stress. This is a major cause of liver damage and egg quality decline (47). The body has an oxidative-antioxidant system. The enzymatic antioxidant system is the most important part. It mainly includes GSH-Px, SOD, and CAT (48). Moderate ROS levels are crucial for cell metabolism, differentiation, transcription factor regulation, and immune defense (49). However, when ROS exceeds the body’s antioxidant capacity, oxidative stress occurs (20). GSH-Px catalyzes the reduction of hydrogen peroxide and organic peroxides into water and stable compounds, inhibiting free radical generation (50). SOD converts superoxide radicals into hydrogen peroxide, which is then reduced to water by CAT and GSH-Px, mitigating oxidative stress damage to cells (51). CAT reduces the accumulation of hydrogen peroxide in the body. Serum T-AOC reflects the overall antioxidant level of the body. Malondialdehyde (MDA), a product of lipid peroxidation, is a key indicator for assessing lipid peroxidation (52). Increasing the activity and concentration of antioxidant enzymes can effectively alleviate oxidative damage in the body. Carotenoids are a class of potent antioxidants (53). Studies have shown that they can improve the production performance of laying hens (42, 43). Stahl et al. (52) found that carotenoids can scavenge singlet molecular oxygen (^1^O_2_) and peroxyl radicals, acting as effective quenchers of singlet oxygen. It has been found (54) that CX can reduce liver ROS and MDA levels, increase GSH-Px and SOD activity, and alleviate oxidative stress in hepatocytes. Sahin et al. (55) discovered that adding lycopene to the diet of Ross 308 broilers can enhance serum GSH-Px and CAT activity and reduce MDA levels (20, 50, and 100 mg/kg). Tao et al. (56) reported that it can lower serum MDA levels and increase SOD activity in oxidative stress rat models. Palozza et al. (57) found that adding CX to the diet can improve liver SOD activity in mice. Nrf-2 is a transcription factor that regulates the expression of SOD, CAT, and other antioxidants (58). Lycopene, astaxanthin, and CX are all carotenoids. Lycopene increases antioxidant enzymes by activating Nrf2 expression (59, 60). Therefore, CX is predicted to have a similar effect. The results show CX increased serum GSH-Px, SOD, and CAT activity in chickens. It also reduced MDA levels. At weeks 3 and 9, 10 mg/kg CX worked best. It boosted GSH-Px, SOD, and CAT activity. At week 3, 6 mg/kg CX improved serum T-AOC most effectively. At weeks 6 and 9, 10 mg/kg CX was optimal. The net increase in antioxidant enzyme activity was positive at all stages. At weeks 6 and 9, 8 mg/kg CX significantly lowered serum MDA levels. These findings suggest CX has a positive effect over time. It also shows a cumulative effect in the body. The mechanism of CX’s antioxidant action is not fully understood. It may enhance GSH-Px, SOD, and CAT activity by increasing Nrf2 expression. It might also suppress NF-κB expression. This could improve T-AOC levels. It may enhance antioxidant capacity in laying hens’ serum. It could reduce MDA levels. It might alleviate liver oxidative stress. It may promote liver TG and TC levels. It could increase serum TG, TC, and HDL-C content. It might lower LDL-C content. It has been found (13) that the addition of 6 mg/kg CX to the diet of laying hens increased SOD and GSH-Px activity in serum and ovarian tissue. This maintained reproductive hormone levels. It promoted ovulation. It improved egg production rate. These results align with the current study.

To clarify the mechanism by CX modulates lipid synthesis, we examined key regulatory genes. Hepatic lipid synthesis is regulated by enzymes including acetyl-CoA carboxylase (ACC), fatty acid synthase (FASN), and malic enzyme (ME), along with transcription factors. In the liver, ACC catalyzes the conversion of acetyl-CoA to malonyl-CoA, initiating *de novo* fatty acid synthesis, with ACC acting as the central regulator of this pathway (61). NADPH required for fatty acid synthesis is provided by the pyruvate-malate cycle (62). ME facilitates the conversion of malate to pyruvate, while FASN governs the rate of fatty acid synthesis. ACC, FASN, and ME are pivotal enzymes for TG and TC synthesis, and their collective activity positively correlates with lipid synthesis rates (63). The sterol regulatory element-binding protein (SREBP) family mainly includes SREBP-1c, SREBP-1a, and SREBP-2. Among them, SREBP-1a/−2 participate in TC metabolism, whereas SREBP-1c, the predominant isoform, transcriptionally regulates hepatic lipid synthesis enzyme genes (64, 65). ACC and FASN are directly controlled by SREBP-1c (66, 67). Additionally, hepatic X-*α* receptor (LXRα) forms a positive feedback loop with SREBP-1c to coordinately regulate hepatic lipogenesis (68). Yeudall et al. (61) demonstrated that ACC knockout disrupts de novo lipogenesis and TC production in murine livers. Xie et al. (67) further observed that reduced expression of ACC, FASN, and SREBP-1c in broiler livers markedly suppresses fatty acid and lipid synthesis. The results of this experiment indicate that the addition of CX to the diet significantly upregulated the expression levels of SREBP-1c, FASN, and ACC genes throughout the entire trial period. This suggests that CX consistently promoted the expression of these genes during the trial, demonstrating a cumulative effect and exhibiting a positive regulatory function in the organism. For LXRα and ME genes, the addition of 4 mg/kg CX at the 6th week of the trial was most effective in enhancing their expression levels. During each phase of the experiment, the changes in gene expression under the optimal dosage showed an initial increase followed by a decrease, without a cumulative effect, indicating that the role of CX is more pronounced during specific periods.

Currently, there are limited reports on the effects of dietary CX supplementation on lipid metabolism in chickens during the laying period. Lycopene, which belongs to the carotenoid family along with CX (69), provides a reference for understanding the mechanism of CX. Liu et al. (70) found that lycopene supplementation stimulates the expression of genes related to fat synthesis and decomposition (such as FASN, ACC, SREBP-1c, DGAT1, and DGAT2) in mice. Cao et al. (71)demonstrated that maternal lycopene supplementation in rats reduces oxidative stress levels in offspring. Furthermore, Moran et al. (72) discovered that lycopene can regulate lipid metabolism by upregulating FASN expression, thereby influencing the process of cellular carcinogenesis. Based on these studies, it is hypothesized that CX may enhance the expression of SREBP-1c and LXRα genes, upregulate hepatic lipid synthase-related genes (such as FASN, ACC, and ME), promote lipid synthesis and utilization, and ensure the transport of lipid nutrients to oocytes, supporting yolk formation and lipid deposition. This mechanism provides a new perspective for understanding how CX regulates hepatic lipid metabolism in chickens.

## 5 Conclusion

The results of this experimental study indicate that dietary supplementation of CX alleviates oxidative stress, enhances egg production, and mitigates liver injury in local chickens by regulating hepatic lipid metabolism. Specifically, CX alleviates hepatic lipid metabolism disorders by enhancing antioxidant capacity, repairing hepatic tissue damage, and upregulating the expression of lipid synthesis-related genes (FASN, ACC, ME, SREBP-1c, and LXRα). This ensures efficient transport of lipid nutrients to oocytes, promotes yolk formation and lipid deposition, and ultimately improves egg production rates. The recommended additive dosage is 8 mg/kg. These findings provide a critical theoretical foundation for applying CX to treat oxidative stress-induced hepatic lipid metabolism disorders and reduced egg production in poultry.

## Data Availability

The original contributions presented in the study are included in the article/Supplementary material, further inquiries can be directed to the corresponding author/s.
