# Supplementary material for: Dietary canthaxanthin improves egg production rate through regulating hepatic lipid metabolism and redox status in indigenous chickens
Source: Front Vet Sci. 2025 Jun 5;12:1607039. doi: 10.3389/fvets.2025.1607039 (PMC12177218; doi:10.3389/fvets.2025.1607039)

Supplementary Material

# **1**Supplementary Data

Supplementary Material should be uploaded separately on submission. Please include any supplementary data, figures and/or tables.

Supplementary material is not typeset so please ensure that all information is clearly presented, the appropriate caption is included in the file and not in the manuscript, and that the style conforms to the rest of the article.

# **2**Supplementary Figures and Tables

**TABLE 1** | Test groups

| **Group** | **Abbreviation** | **Daily ration** |
| --- | --- | --- |
| control group | NC | Basic diet |
| Adding Group 1 | NT1 | Basic diet +4 mg/kg CX |
| Adding Group 2 | NT2 | Basic diet +6 mg/kg CX |
| Adding Group 3 | NT3 | Basic diet +8 mg/kg CX |
| Adding Group 4 | NT4 | Basic diet +10 mg/kg CX |

**TABLE 2** | Basal diet composition and nutrient level (air-dried basis)

| **Items** | **Content** |
| --- | --- |
| Corn | 55.00 |
| Soybean meal | 20.00 |
| Wheat bran | 9.50 |
| Fish meal | 5.00 |
| Limestone | 7.50 |
| CaHPO4 | 2.50 |
| NaCl | 0.10 |
| Premix ^1)^ | 0.40 |
| Total | 100.00 |
| ME /(MJ/kg) ^2^ | 11.60 |
| CP | 15.50 |
| Ca | 2.0 |
| TP | 0.63 |
| Met | 0.40 |
| Cys | 0.30 |
| Lys | 0.80 |

Note：The nutrient levels are calculated values. The premix provided per kg of diet: VA 9000 IU, VD 2500 IU, VE 20 IU, VB 1212 μg, VK 2.4 mg; Mn100 mg, Zn 60 mg, Fe 25 mg, Cu 5 mg, Co 0.1 mg (Mn、Zn、Fe、Cu、Co are provided in the form of sulphate), Se (N2SeO3•5H2O) 0.2 mg, I (KI) 0.5 mg. All values are measured except metabolizable energy.

**TABLE 3** | Primers for real-time PCR

| **Genes** | **Primer Sequence (5'-3')** | **Product Size (bp)** |
| --- | --- | --- |
| SREBP-1c | F: GCCCTCTGTGCCTTTGTCTTC  R: ACTCAGCCATGATGCTTCTTCC | 130 |
| ACACA | F: AATGGCAGCTTTGGAGGTGT  R: TCTGTTTGGGTGGGAGGTG | 136 |
| FASN | F: CGCAGTTTGTTGATGGTGAG  R: TCCTTGGTGTTCGTGACG | 179 |
| LXRα | F: GTCCCTGACCCTAATAACCGC  R: GTCTCCAACAACATCACCTCTATG | 186 |
| ME | F: TGCCAGCATTACGGTTTAGC  R: CCATTCCATAACAGCCAAGGTC | 175 |
| *β*-actin | F: CAACACAGTGCTGTCTGGTGGTAC  R: CTCCTGCTTGCTGATCCACATCTG | 199 |
| SREBP-1c, sterol regulatory element-binding protein 1c; ACACA, Ace-CoA carboxylase; FASN, fatty acidsynthase; LXRα, liver X receptor α; ME, malic enzyme. | | |

**TABLE 4** | Effect of dietary supplementation with graded levels of canthaxanthin (CX) on liver index, liver weight and egg production rate of laying chickens.

| **Item** | **Week** | **NC** | **NT1** | **NT2** | **NT3** | **NT4** | ***P*-value** | **SEM** | ***P*-value** | | |
| --- | --- | --- | --- | --- | --- | --- | --- | --- | --- | --- | --- |
|  |  |  |  |  |  |  |  |  | **Period** | **CX** | **Period × CX** |
| Liver weight/g | 3 weeks | 30.51^AB^ | 28.83^B^ | 37.17^AB^ | 41.20^Aa^ | 26.77^Bb^ | 0.09 | 4.04 | 0.48 | 0.55 | ＜0.05 |
|  | 6 weeks | 36.03 | 34.67 | 28.67 | 35.00^ab^ | 35.67^ab^ | 0.69 |  |  |  |  |
|  | 9 weeks | 27.67^B^ | 26.67^B^ | 31.33^B^ | 25.67^Bb^ | 43.30^Aa^ | ＜0.05 |  |  |  |  |
|  | *P*-value | 0.34 | 0.36 | 0.33 | ＜0.05 | ＜0.05 |  |  |  |  |  |
| Liver index/% | 3 weeks | 1.48 | 1.22 | 1.56 | 1.77 | 1.23 | 0.23 | 0.19 | 0.07 | 0.82 | 0.17 |
|  | 6 weeks | 1.71 | 1.88 | 1.62 | 1.8 | 1.65 | 0.86 |  |  |  |  |
|  | 9 weeks | 1.56 | 1.43 | 1.81 | 1.46 | 2.03 | 0.16 |  |  |  |  |
|  | *P*-value | 0.69 | 0.06 | 0.62 | 0.38 | 0.02 |  |  |  |  |  |
| Egg production rate% | 3 weeks | 43.28^ABc^ | 43.64^ABb^ | 46.20^Ac^ | 47.89^Ac^ | 39.17^Bb^ | ＜0.05 | 1.77 | ＜0.05 | ＜0.05 | ＜0.05 |
|  | 6 weeks | 49.48^Bb^ | 48.06^Bb^ | 55.34^Ab^ | 55.70^Ab^ | 39.75^Cb^ | ＜0.05 |  |  |  |  |
|  | 9 weeks | 56.09^Ba^ | 68.77^Aa^ | 69.89^Aa^ | 67.07^Aa^ | 55.34^Ba^ | ＜0.05 |  |  |  |  |
|  | *P*-value | ＜0.05 | ＜0.05 | ＜0.05 | ＜0.05 | ＜0.05 |  |  |  |  |  |
| Note: Uppercase letters indicate differences between different dose groups within the same period, while lowercase letters indicate differences within the same dose group across different periods. Data labels without letters or with the same letters indicate no significant difference (*P* > 0.05), whereas different lowercase or uppercase letters denote significant differences (*P* < 0.05). | | | | | | | | | | | |

**TABLE 5** | Effect of dietary supplementation with graded levels of canthaxanthin (CX) on serum and liver lipid content of laying chickens.

| **Item** | **Week** | **NC** | **NT1** | **NT2** | **NT3** | **NT4** | ***P*-value** | **SEM** | ***P*-value** | | |
| --- | --- | --- | --- | --- | --- | --- | --- | --- | --- | --- | --- |
|  |  |  |  |  |  |  |  |  | **Period** | **CX** | **Period × CX** |
| Liver TG/mmol/L | 3 weeks | 6.45^Bb^ | 7.74^Ac^ | 6.34^Bb^ | 6.3219^Bb^ | 5.97^Bb^ | ＜0.05 | 0.32 | ＜0.05 | ＜0.05 | ＜0.05 |
|  | 6 weeks | 6.32^Bb^ | 10.96^Ab^ | 11.17^Ab^ | 10.49^Aa^ | 10.85^Aa^ | ＜0.05 |  |  |  |  |
|  | 9 weeks | 10.79^ABa^ | 9.01^Ca^ | 11.43^Ab^ | 10.05^Ba^ | 10.69^ABa^ | ＜0.05 |  |  |  |  |
|  | *P*-value | ＜0.05 | ＜0.05 | ＜0.05 | ＜0.05 | ＜0.05 |  |  |  |  |  |
| Liver TC/mmol/L | 3 weeks | 81.30^Ac^ | 59.59^Bc^ | 53.11^Bc^ | 53.58^Bc^ | 50.35^Bc^ | ＜0.05 | 4.22 | ＜0.05 | 0.07 | ＜0.05 |
|  | 6 weeks | 125.75^Cb^ | 124.06^Cb^ | 159.30^Bb^ | 157.53^Ba^ | 171.34^Aa^ | ＜0.05 |  |  |  |  |
|  | 9 weeks | 153.85^ABa^ | 150.663^Aa^ | 160.61^Aa^ | 156.05^Ab^ | 142.82^Bb^ | 0.05 |  |  |  |  |
|  | *P*-value | ＜0.05 | ＜0.05 | ＜0.05 | ＜0.05 | ＜0.05 |  |  |  |  |  |
| Serum TG/mmol/L | 3 weeks | 2.33^a^ | 2.36^b^ | 2.22^b^ | 2.21^b^ | 2.39^b^ | 0.47 | 0.08 | ＜0.05 | ＜0.05 | ＜0.05 |
|  | 6 weeks | 1.98^Cb^ | 2.67^Ba^ | 2.63^Ba^ | 3.05^Aa^ | 2.99^Aa^ | ＜0.05 |  |  |  |  |
|  | 9 weeks | 1.88^Cb^ | 2.69^ABa^ | 2.58^Ba^ | 2.82^Aa^ | 2.83^Aa^ | ＜0.05 |  |  |  |  |
|  | *P*-value | ＜0.05 | ＜0.05 | ＜0.05 | ＜0.05 | ＜0.05 |  |  |  |  |  |
| Serum TC/mmol/mL | 3 weeks | 5.55 | 5.47^b^ | 5.65^b^ | 5.08^b^ | 5.31^b^ | 0.57 | 0.26 | ＜0.05 | ＜0.05 | ＜0.05 |
|  | 6 weeks | 6.28^C^ | 8.61^Aa^ | 7.64^Ba^ | 8.20^ABa^ | 7.60^Ba^ | ＜0.05 |  |  |  |  |
|  | 9 weeks | 6.22^C^ | 8.53^Aa^ | 7.64^Ba^ | 8.23^ABa^ | 7.48^Ba^ | ＜0.05 |  |  |  |  |
|  | *P*-value | 0.11 | ＜0.05 | ＜0.05 | ＜0.05 | ＜0.05 |  |  |  |  |  |
| Serum LDL-C/μmol/L | 3 weeks | 2.75^Ab^ | 2.19^Ab^ | 2.17^Ab^ | 2.08^Bb^ | 2.92^Ab^ | ＜0.05 | 0.25 | ＜0.05 | ＜0.05 | ＜0.05 |
|  | 6 weeks | 3.55^Aa^ | 3.47^Aa^ | 2.36^Bb^ | 3.15^Aa^ | 3.401^Ab^ | ＜0.05 |  |  |  |  |
|  | 9 weeks | 3.22^Bab^ | 4.00^Aa^ | 3.53^ABa^ | 2.41^Cb^ | 4.09^Aa^ | ＜0.05 |  |  |  |  |
|  | *P*-value | ＜0.05 | ＜0.05 | ＜0.05 | ＜0.05 | ＜0.05 |  |  |  |  |  |
| Serum HDL-C/μmol/L | 3 weeks | 1.77^Aa^ | 1.86^Aa^ | 1.65^Ba^ | 1.77^Aa^ | 1.61^Ba^ | ＜0.05 | ＜0.05 | ＜0.05 | ＜0.05 | ＜0.05 |
|  | 6 weeks | 0.83^Cb^ | 0.97^Bb^ | 1.04^ABb^ | 1.14^Ab^ | 1.04^ABb^ | ＜0.05 |  |  |  |  |
|  | 9 weeks | 0.78^Cb^ | 0.93^Bb^ | 0.99^Bb^ | 1.11^Ab^ | 0.98^Bb^ | ＜0.05 |  |  |  |  |
|  | *P*-value | ＜0.05 | ＜0.05 | ＜0.05 | ＜0.05 | ＜0.05 |  |  |  |  |  |
| Note: Uppercase letters indicate differences between different dose groups within the same period, while lowercase letters indicate differences within the same dose group across different periods. Data labels without letters or with the same letters indicate no significant difference (*P* > 0.05), whereas different lowercase or uppercase letters denote significant differences (*P* < 0.05). | | | | | | | | | | | |

**TABLE 6** | Effect of dietary supplementation with graded levels of canthaxanthin (CX) on serum antioxidant properties of laying chickens.

| **Item** | **Week** | **NC** | **NT1** | **NT2** | **NT3** | **NT4** | ***P*-value** | **SEM** | ***P*-value** | | |
| --- | --- | --- | --- | --- | --- | --- | --- | --- | --- | --- | --- |
|  |  |  |  |  |  |  |  |  | **Period** | **CX** | **Period × CX** |
| T-AOC(μmolTrolox/g ） | 3 weeks | 3.55^ABa^ | 3.23^Bb^ | 3.59^Ab^ | 3.59^Ab^ | 3.38^Ab^ | 0.15 | 0.12 | ＜0.05 | ＜0.05 | ＜0.05 |
|  | 6 weeks | 2.78^Bb^ | 4.134^Aa^ | 4.08^Aa^ | 4.14^Aa^ | 4.32^Aa^ | ＜0.05 |  |  |  |  |
|  | 9 weeks | 2.84^Bb^ | 4.06^Aa^ | 4.00^Aa^ | 4.27^Aa^ | 4.31^Aa^ | ＜0.05 |  |  |  |  |
|  | *P*-value | ＜0.05 | ＜0.05 | ＜0.05 | ＜0.05 | ＜0.05 |  |  |  |  |  |
| MDA(nmol/L ） | 3 weeks | 11.70^Bb^ | 13.75^Ab^ | 13.95^Ab^ | 14.32^Ab^ | 14.37^Ab^ | ＜0.05 | 0.36 | ＜0.05 | ＜0.05 | ＜0.05 |
|  | 6 weeks | 20.40^Aa^ | 19.03^Ba^ | 18.20^BCa^ | 17.43^Ca^ | 18.26^BCa^ | ＜0.05 |  |  |  |  |
|  | 9 weeks | 20.39^Aa^ | 19.14^Ba^ | 17.83^CDa^ | 16.97^Da^ | 18.31^BCa^ | ＜0.05 |  |  |  |  |
|  | *P*-value | ＜0.05 | ＜0.05 | ＜0.05 | ＜0.05 | ＜0.05 |  |  |  |  |  |
| CAT(U/mL ） | 3 weeks | 43.65^Da^ | 49.44^Ca^ | 52.07B^Ca^ | 52.61^ABa^ | 55.67^Aa^ | ＜0.05 | 1.08 | ＜0.05 | ＜0.05 | 0.12 |
|  | 6 weeks | 38.00^Cb^ | 43.35^Bb^ | 46.46^ABb^ | 49.14^Ab^ | 45.10^Bb^ | ＜0.05 |  |  |  |  |
|  | 9 weeks | 37.36^Db^ | 43.49^Cb^ | 46.77^ABb^ | 49.21^Ab^ | 45.58^BCb^ | ＜0.05 |  |  |  |  |
|  | *P*-value | ＜0.05 | ＜0.05 | ＜0.05 | ＜0.05 | ＜0.05 |  |  |  |  |  |
| GSH-Px(IU/L） | 3 weeks | 459.53^C^ | 536.39^B^ | 559.70^ABa^ | 559.41^AB^ | 589.31^A^ | ＜0.05 | 15.26 | ＜0.05 | ＜0.05 | 0.63 |
|  | 6 weeks | 412.18^B^ | 537.77^A^ | 507.59^Ab^ | 513.67^A^ | 522.26^A^ | ＜0.05 |  |  |  |  |
|  | 9 weeks | 411.04^B^ | 522.76^A^ | 501.63^Ab^ | 509.32^A^ | 532.01^A^ | ＜0.05 |  |  |  |  |
|  | *P*-value | 0.051 | 0.75 | ＜0.05 | 0.05 | 0.01 |  |  |  |  |  |
| SOD(U/mL） | 3 weeks | 88.21^D^ | 102.43^C^ | 105.15^BC^ | 110.42^AB^ | 113.04^A^ | ＜0.05 | 1.87 | 0.46 | ＜0.05 | 0.26 |
|  | 6 weeks | 84.41^B^ | 105.49^B^ | 107.66^AB^ | 104.27^B^ | 111.55^A^ | ＜0.05 |  |  |  |  |
|  | 9 weeks | 84.15^C^ | 103.84^B^ | 107.80^AB^ | 105.28^B^ | 111.19^A^ | ＜0.05 |  |  |  |  |
|  | *P*-value | 0.25 | 0.52 | 0.54 | 0.06 | 0.76 |  |  |  |  |  |
| Note: Uppercase letters indicate differences between different dose groups within the same period, while lowercase letters indicate differences within the same dose group across different periods. Data labels without letters or with the same letters indicate no significant difference (*P* > 0.05), whereas different lowercase or uppercase letters denote significant differences (*P* < 0.05). | | | | | | | | | | | |

## **2.1** Supplementary Figures


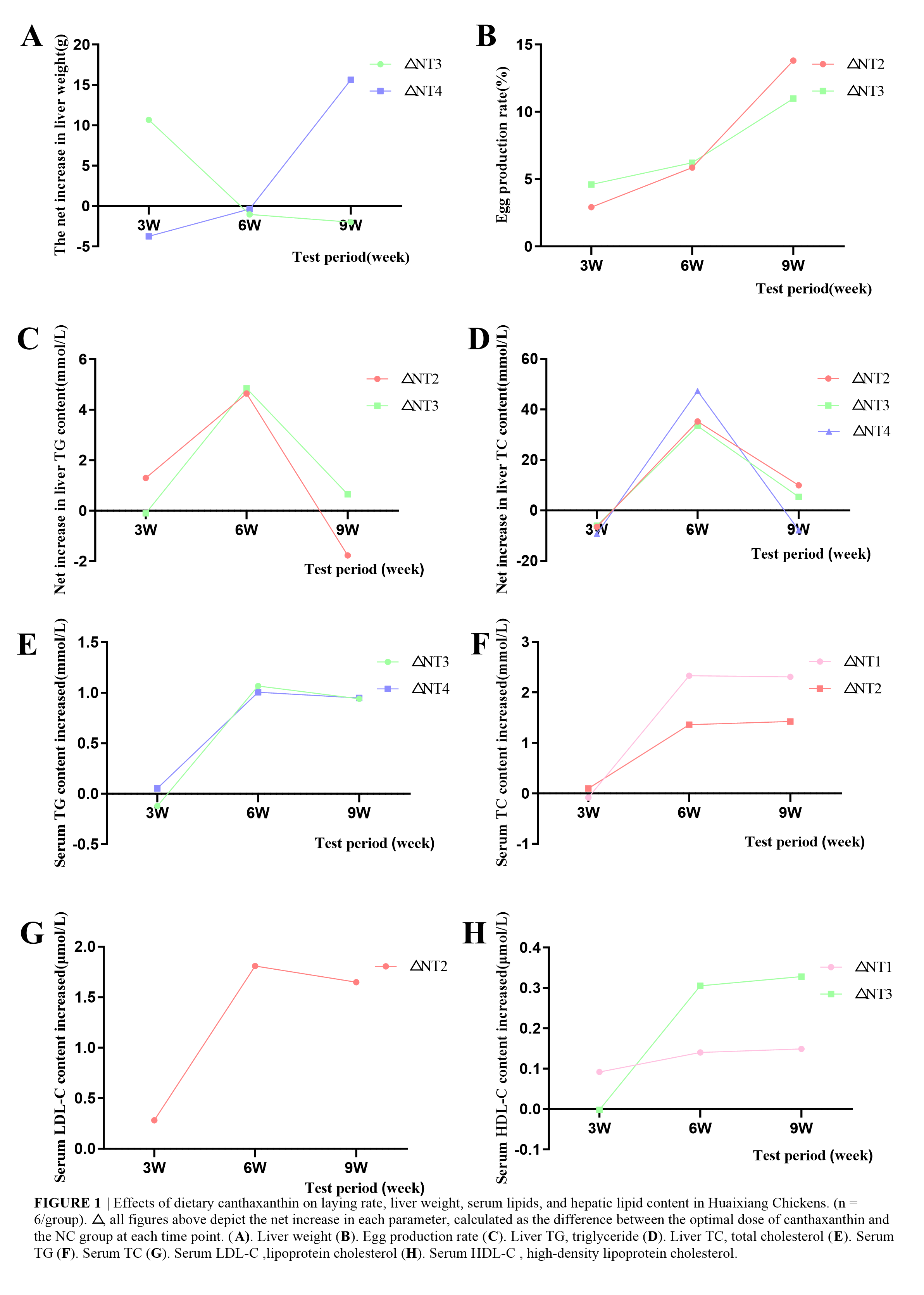


**
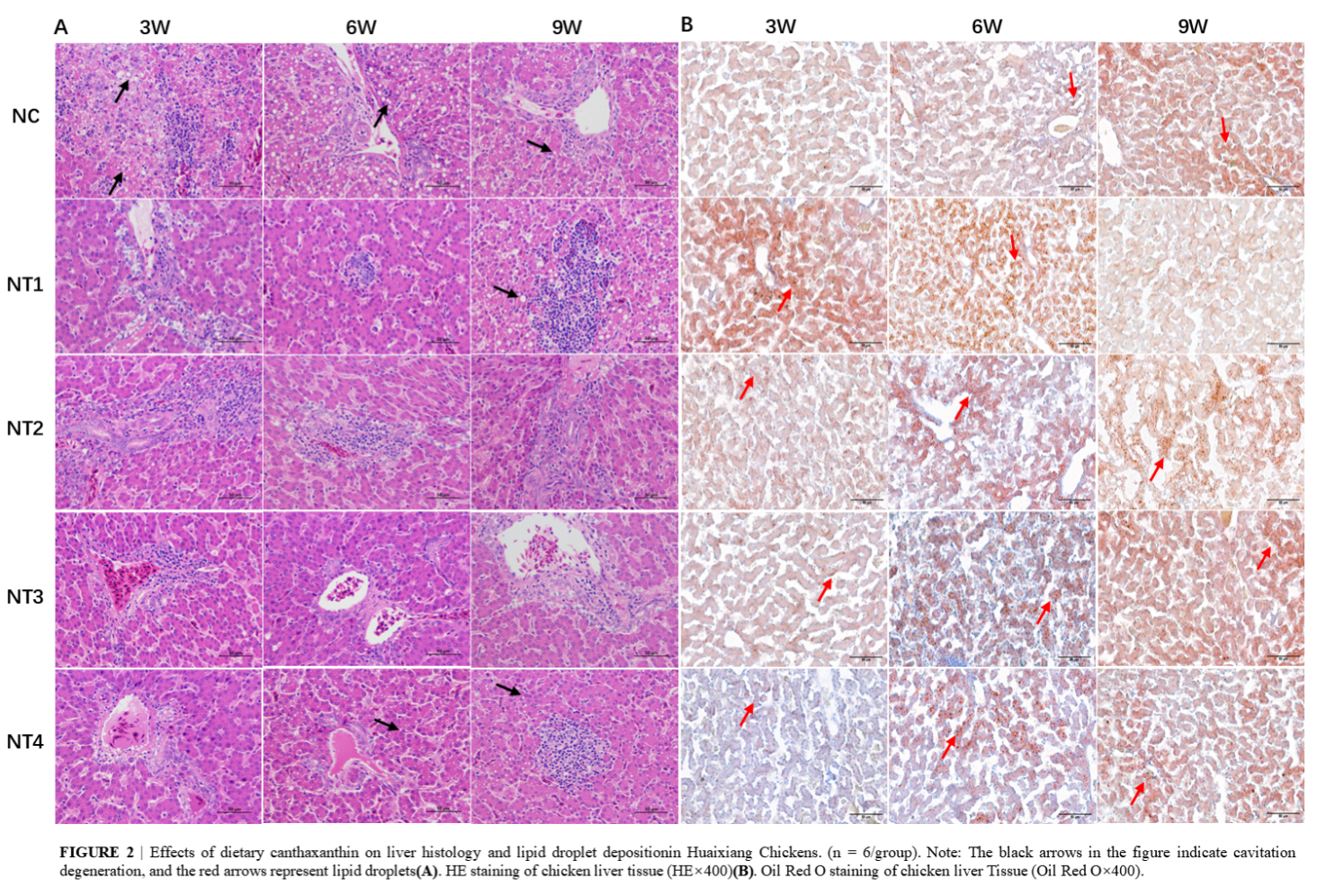
**

**
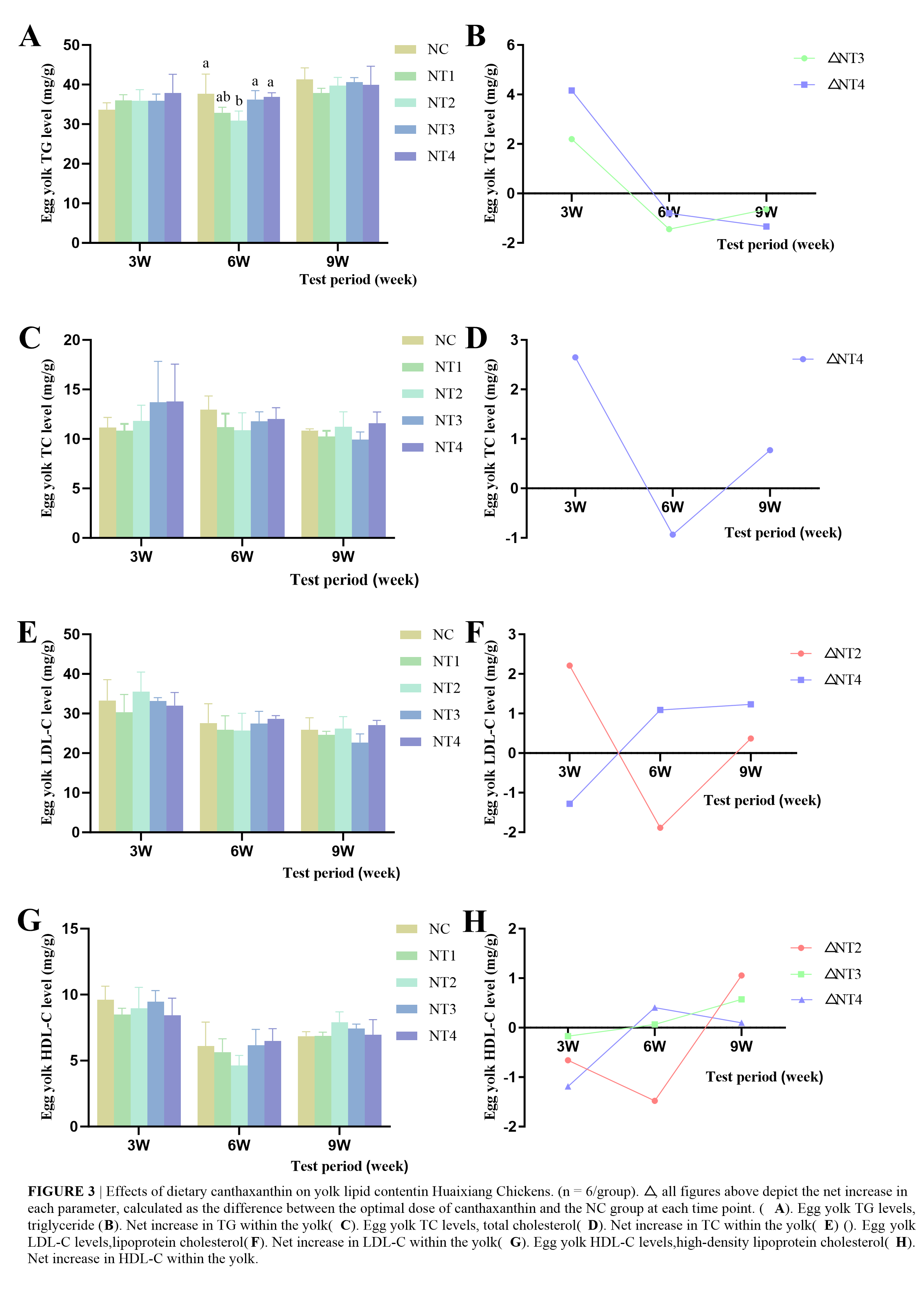
**


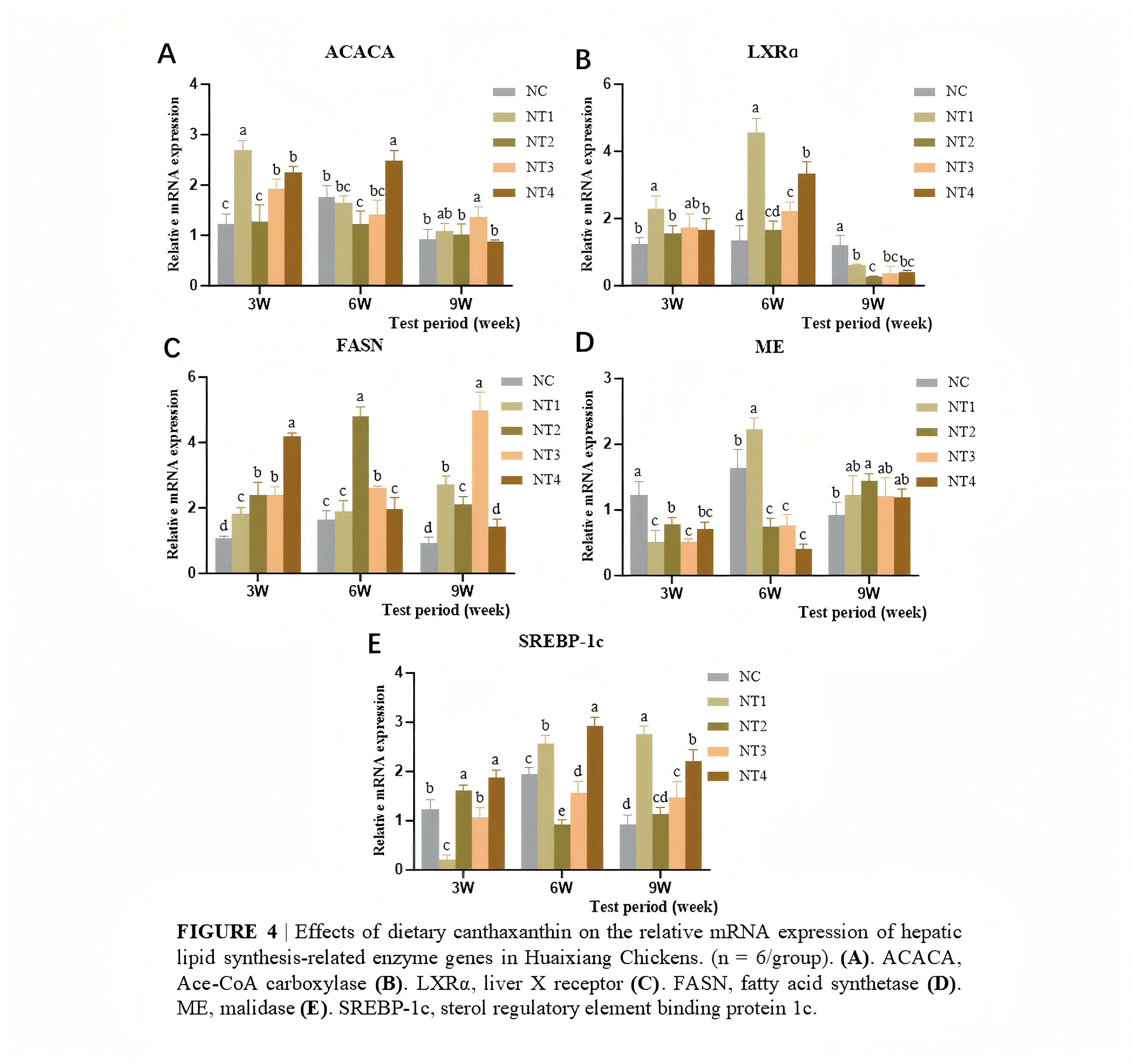

Supplement: Supplementary file 1 [file Data_Sheet_1.docx]
